# Diethylcarbamazine, TRP channels and Ca^2+^ signaling in cells of the *Ascaris* intestine

**DOI:** 10.1038/s41598-022-25648-7

**Published:** 2022-12-09

**Authors:** Paul D. E. Williams, Sudhanva S. Kashyap, Mark A. McHugh, Matthew T. Brewer, Alan P. Robertson, Richard J. Martin

**Affiliations:** 1 Department of Biomedical Sciences, Iowa State University, Ames, IA USA; 2 Department of Veterinary Pathology, Iowa State University, Ames, IA USA

**Keywords:** Biological techniques, Drug discovery, Molecular biology, Zoology, Diseases, Medical research

## Abstract

The nematode parasite intestine absorbs nutrients, is involved in innate immunity, can metabolize xenobiotics and as we show here, is also a site of action of the anthelmintic, diethylcarbamazine. Diethylcarbamazine (DEC) is used to treat lymphatic filariasis and activates TRP-2, GON-2 & CED-11 TRP channels in *Brugia malayi* muscle cells producing spastic paralysis. DEC also has stimulatory effects on ascarid nematode parasites. Using PCR techniques, we detected, in *Ascaris suum* intestine, message for: *Asu-trp-2, Asu-gon-2, Asu-ced-11, Asu-ocr-1, Asu-osm-9* and *Asu-trpa-1.* Comparison of amino-acid sequences of the TRP channels of *B. malayi,* and *A. suum* revealed noteworthy similarity, suggesting that the intestine of *Ascaris* will also be sensitive to DEC. We used Fluo-3AM as a Ca^2+^ indicator and observed characteristic unsteady time-dependent increases in the Ca^2+^ signal in the intestine in response to DEC. Application of La^3+^ and the TRP channel inhibitors, 2-APB or SKF 96365, inhibited DEC mediated increases in intracellular Ca^2+^. These observations are important because they emphasize that the nematode intestine, in addition to muscle, is a site of action of DEC as well as other anthelmintics. DEC may also enhance the Ca^2+^ toxicity effects of other anthelmintics acting on the intestine or, increase the effects of other anthelmintics that are metabolized and excreted by the nematode intestine.

## Introduction

Soil-transmitted helminths (STHs), including *Ascaris, Trichuris,* and hookworm, are a primary medical and public health concern in many developing countries. It is estimated that approximately 807 million to 1.2 billion people are infected with *Ascaris lumbricoides*^1^. Although not usually fatal, parasitic infections have a detrimental effect on morbidity, reducing worker productivity by 6.3 million **D**isability **A**djusted **L**ife **Y**ears (DALYs) per year. Parasitic infections affect worker health, output, and the performance of children in school. In livestock, infestations lead to reduced food yields, impacting economic returns and exacerbating poverty^2^.

Currently, there are no effective vaccines against STH infections, and treatment relies on the use of chemotherapeutics, usually using one of the three major classes of anthelmintics: benzimidazoles (albendazole/mebendazole), macrocyclic lactones (ivermectin), or nicotinic cholinergics (levamisole and pyrantel). There is a risk of resistance developing in humans associated with regular use of these compounds, as seen in animal parasites^3^. With the limited number of anthelmintic drugs and the risk of resistance development, identifying the modes of action of other existing anthelmintics and predicting rational anthelmintic combinations, which are more potent and effective, is anticipated to counter the resistance.

The classic drug diethylcarbamazine (DEC) is used to treat lymphatic filariasis caused by filarial parasites including *Brugia malayi* and *Wucheria bancrofti*^4,5^, but DEC has also been used to treat STH infections, including ascariasis^6^. Although DEC has been used for over seventy years, its mode of action is poorly understood. DEC's recently discovered actions are to open nematode muscle Transient Receptor Potential (TRP) channels, including the TRPMs, GON-2 & CED-11, and the TRPC, TRP-2^7^. The opening of muscle cell TRPs allows entry of Ca^2+^ and transient spastic paralysis. TRP channels in nematodes are not only found in muscle cells, but they are also found distributed throughout the different tissues of nematodes including the intestine^8,9^. This suggests that DEC will also affect the cells of the intestine and impair its vital functions.

The nematode intestine is a long tube of single-layer columnar epithelial cells that connects the base of the pharynx to the anus. The intestine is vital for nematode survival. It is essential for digestion and the transport of ion solutes and nutrients^10–14^. It is involved in defense against environmental toxins and microbial infection by mediating innate immunity responses and the release of antimicrobial peptides^15,16^. The intestine is also a major site of metabolism of xenobiotic drugs by cytochrome P450 oxidative enzymes^17^: it excretes xenobiotic compounds and their metabolites (including anthelmintic drugs) via its numerous ABC transporters and organic anion transporters^18^. Toxic effects on the nematode intestine will produce a cascade of damage on other tissues including the neuromuscular and reproductive tissues of the parasite.

The intestine of parasitic nematodes is also one of the sites of action of anthelmintic drugs. The benzimidazoles, like mebendazole, disrupt the microtubules and block the transport of secretory granules and movement of subcellular organelles in the intestine of *Ascaris suum*^19^. Levamisole, a cholinergic anthelmintic, targets nicotinic ion-channel receptors that are present in muscle^20^ and intestinal cells of *A. suum*^21^. Another potential anthelmintic acting on the intestine is Cry5B, a Cry protein recovered from *Bacillus thuringiensis,* which forms pores in the intestine membranes^22–26^. Cry5B has potent toxic effects on a range of parasitic nematodes, including hookworm, *Strongyloides* and *A. suum*^26–28^*.*

Here we report the effects of DEC on the intestine of the pig worm, *Ascaris suum,* which is genetically the same species as the human worm, *Ascaris lumbricoides*^29,30^. We used Ca^2+^ imaging to record simultaneously from groups of intestine cells in isolated open intestine flap preparations*.* This allowed us to separate and characterize the effects of anthelmintics on the intestine from the underlying muscle and hypodermis of the worm. We identified the message of different types of TRP channels in the intestine to compare it with the underlying muscle bags of the parasite and found different levels of tissue expression. The Ca^2+^ imaging also showed us that DEC produces an increase in intracellular Ca^2+^ in the intestine by opening TRP channels that allow entry of extracellular Ca^2+^ but DEC did not mediate an intracellular release of Ca^2+^. The rise in the cytoplasmic Ca^2+^ will be toxic and limit normal functions, including metabolism and excretion of xenobiotic drugs. By this mechanism, DEC is predicted to synergize with and enhance the effects of other anthelmintics.

## Results

### TRP channels in *Ascaris*

**Figure 1:**
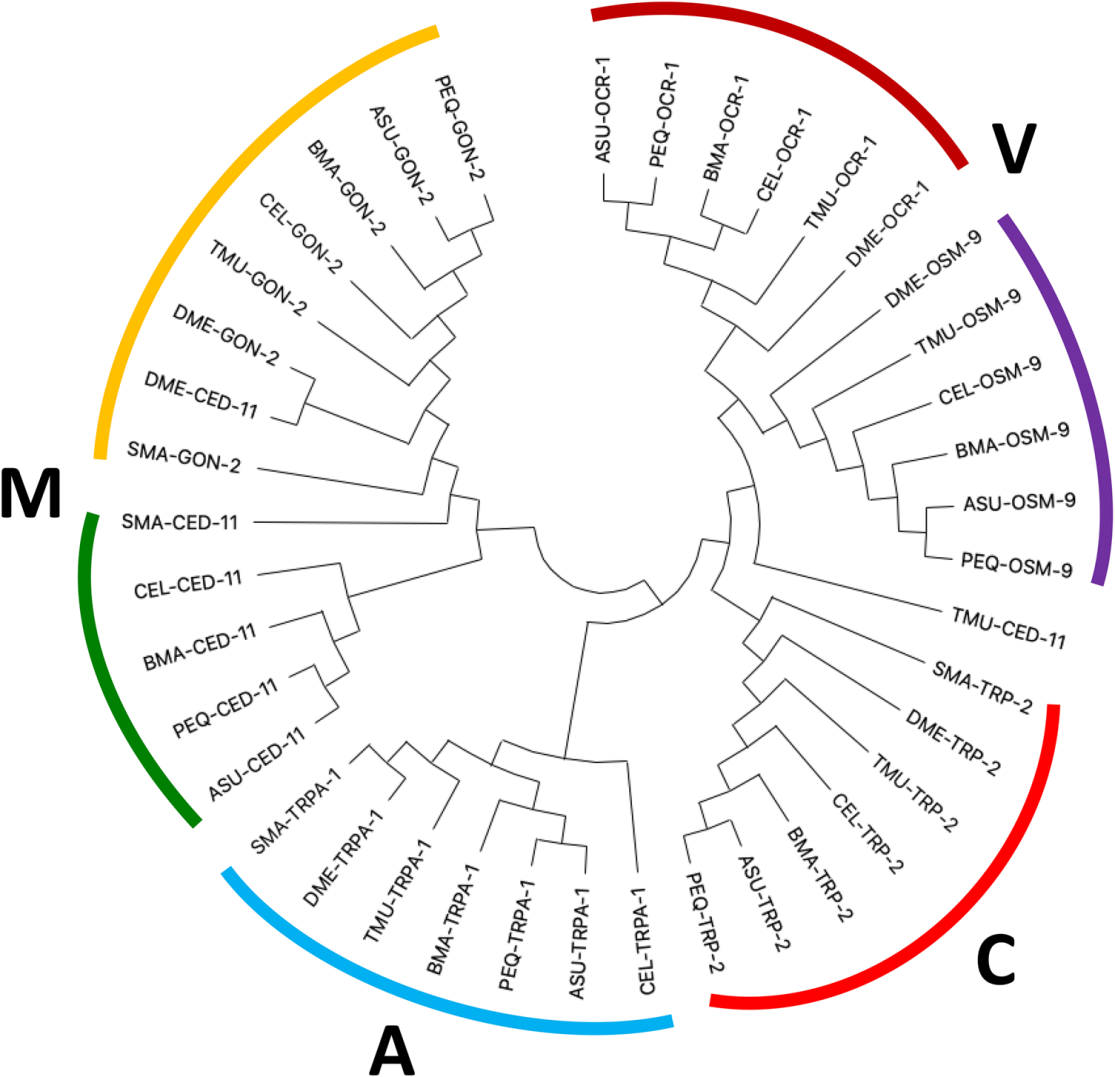
Phylogenetic of TRP channels protein sequences conservation across multiple parasitic species showing that Clase III, *Ascaris suum, Parascaris equorum* and *Brugia malayi* are similar: Phylogenetic tree showing conservation of the TRPM channels GON-2 and CED-11, TRPC channel TRP-2, TRPV channels OCR-1 and OSM-9 and TRPA channel TRPA-1 in multiple species; Clade III *Ascaris suum* (ASU)*, Parascaris equorum* (PEQ) and *Brugia malayi* (BMA), Clade V *Caenorhabditis. elegans* (CEL), Clade I *Trichuris muris* (TMU), schistosome, *Schistosoma mansoni* (SMA) and the fly *Drosophila melanogaster* (DME). The tree was *constructed* using MEGA X software by maximum likelihood method based on the Le and Gascuel model.

In the filarial nematode, *Brugia malayi,* DEC stimulates spastic muscle paralysis by opening muscle TRP channels of the TRPM group (*Bma*-GON-2 and *Bma*-CED-11) and the TRPC group (*Bma*-TRP-2)^7^. We blasted these genes against the *A. suum* (ASU) database (Wormbase) and found *Asu-gon-2, Asu-ced-11*, *Asu-trp-2*. We also found the TRPV genes, *Asu-ocr-1* & *Asu-osm-9*, and the TRPA channel gene, *Asu-trpa-1*. We compared the sequences of these six TRP *A. suum* genes: *Asu-gon-2, Asu-ced-11, Asu-trp-2, Asu-ocr-1, Asu-osm-9,* and *Asu-trpa-1* with the sequences of the same genes in: (1) the Clade III nematode parasites, *Parascaris equorum* (PEQ) and *Brugia malayi* (BMA); (2) the Clade V nematode, *C. elegans* (CEL); (3) the Clade I nematode parasite, *Trichuris muris* (TMU); (4) the schistosome, *Schistosoma mansoni* (SMA) and; (5) the fly, *Drosophila melanogaster* (DME)*.* Accession number links to all sequences used can be found in Supplementary TableS3*.* The dendrogram, Fig. 1, shows that the TRP channels of *A. suum* and *B. malayi* are more closely related than to the TRP genes of *C. elegans, S. mansoni,* and *D. melanogaster.* The close relationship of the TRP channels of *A. suum* and *B. malayi* encouraged the hypothesis that TRP channels of *A. suum* and *B. malayi* have a similar pharmacology and sensitivity to DEC.

### TRP channel expression in *Ascaris* intestine

**Figure 2:**
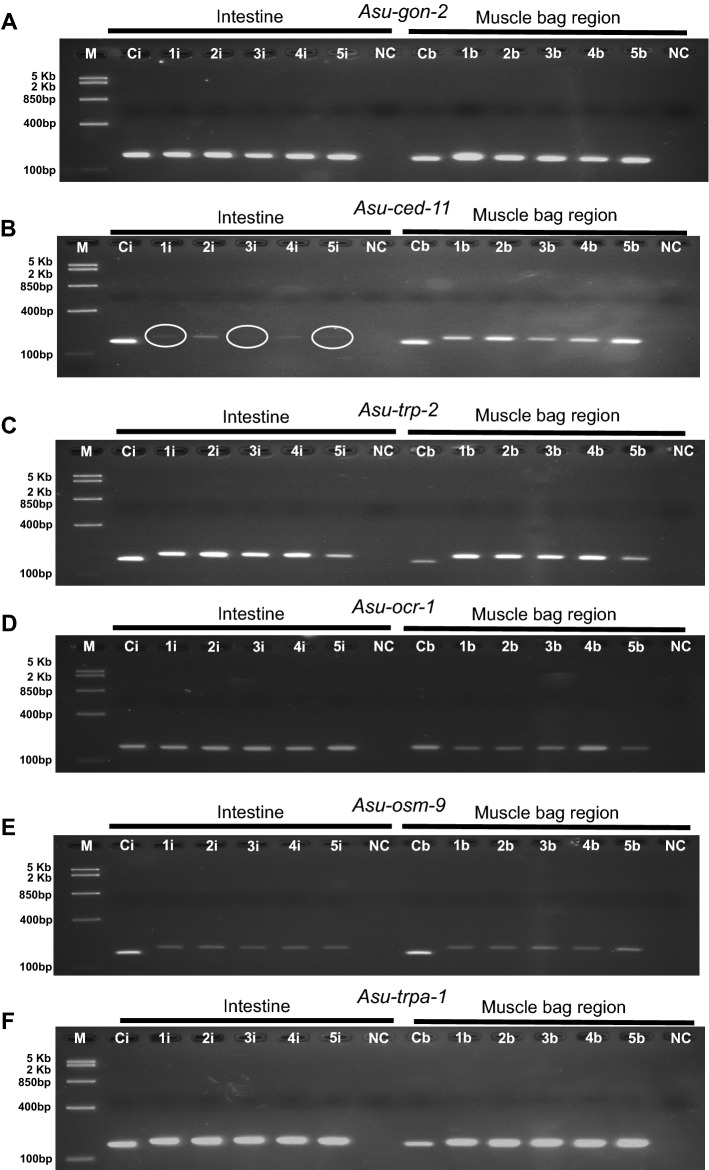
Localization of TRP channels in the intestine and muscle bag region. RT-PCR analysis of intestine (1i, 2i, 3i, 4i, 5i) and muscle bag (1b, 2b, 3b, 4b, 5b) of five separate female *A. suum* worms. Each lane represents the intestine or muscle bag of an individual worm. *Asu-gapdh* from the intestine (Ci) or muscle bag (Cb) was used as a positive control. N.C. = negative control, no cDNA template present. M = Fast Ruler Middle Range DNA Ladder (ThermoFisher Scientific). (**A**) *Asu-gon-2*, (**B**) *Asu-ced-11* (**C**) *Asu-trp-2*, (**D**) *Asu-ocr-1* (**E**) *Asu-osm-9*, (**F**) *Asu-trpa-1*. Note the reduced intensity of the bands (white circles) with *Asu-ced-11* from the intestine. All images are cropped. Images were taken under UV light with an exposure setting of 3 s per 1 frame. Original gel images are presented in Supplementary Fig. S1.

To determine if these TRP channel genes are expressed in the intestine and muscle bags of *Ascaris,* we generated screening primers targeting the coding region of these six genes (Supplementary Table S1). We compared the expression found in the intestine and expression found in the muscle bag regions of the same animal. We found that all six genes were expressed in both the intestine and the muscle bag regions (Fig. 2; for original uncropped gel pictures see Supplementary Fig. S1) although, their band intensities varied between muscle and intestine. Thus, the TRP channels (*Asu-*GON-2, *Asu-*CED-11 & *Asu-*TRP-2) that are associated with mediating DEC effects on the muscle of the filarial parasite, *Brugia malayi,* are similar in sequence and present in the intestine of *A. suum*.

### TRP channels have tissue-specific expressions levels in *Ascaris*

Variations in band intensity for the different TRP channels between the intestine and muscle bag regions suggested differences in the relative mRNA expression levels for each channel. We used qPCR to measure the mRNA expression levels for each of the six TRP channels in the intestine relative to the reference gene, *Asu-gapdh*. We compared the TRP channel expression levels to paired muscle bags from the same *Ascaris* parasite.

**Figure 3:**
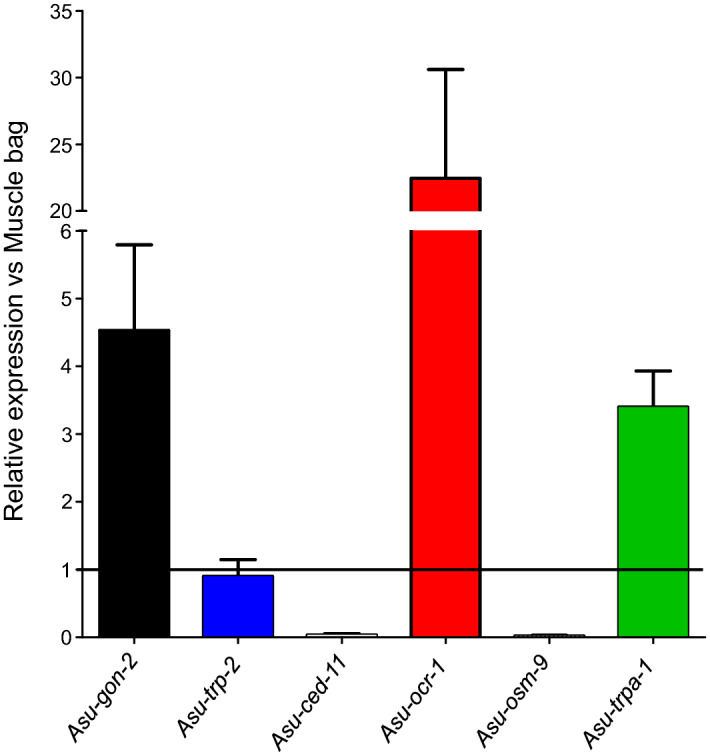
Differential expression of TRP channels between intestine and muscle bag. Bar charts (expressed as mean ± SEM) demonstrating transcript level analysis for TRP channels from intestinal samples for GON-2 (black bar), TRP-2 (blue bar), CED-11 (small), OCR-1 (red bar), OSM-9 (small) and TRPA-1 (green bar) when compared to paired muscle samples. *n* = 15 individual worms over three biological replicates.

Our analysis showed that *Asu-gon-2* had a higher expression level in the intestine compared to the muscle bags (4.5-fold, Fig. 3, black bar). We observed that *Asu-trp-2* had similar levels of expression in both the intestine and muscle bag (Fig. 3, blue bar). For *Asu-ced-11,* we observed a reduced expression level in the intestine compared to muscle, suggesting that the CED-11 function in the intestine is less significant, Fig. 3. For the TRPV channels, we observed that OCR-1 has a higher expression level in the intestine, (20-fold, Fig. 3, red bar), but that OSM-9 expression was relatively low in the intestine (Fig. 3). Lastly, for TRPA-1, qPCR showed relatively high levels of expression in the intestine (3.5-fold) compared to the muscle (Fig. 3, green bar). The different levels of expressions of the different TRP channel transcripts will relate to the different physiological functions of the *A. suum* cells of the intestine and cells of the muscles.

In *B. malayi,* DEC activates TRP channels that involve TRP-2, GON-2, and CED-11 subunits. The presence of clear and significant expression levels of *Asu-gon-2* and similar expression of *Asu-trp-2* but not *Asu-ced-11* suggests that the GON-2 and TRP-2 channels are activated by DEC in the *Ascaris* intestine. We do not exclude possible effects of DEC on the TRPV channel, OCR-1, and the TRPA channel, TRPA-1, in the intestine of *A. suum* which have high levels of expression in the intestine.

### DEC stimulates a prolonged Ca^2+^ signal in the *Ascaris* intestine via TRP channels

**Figure 4:**
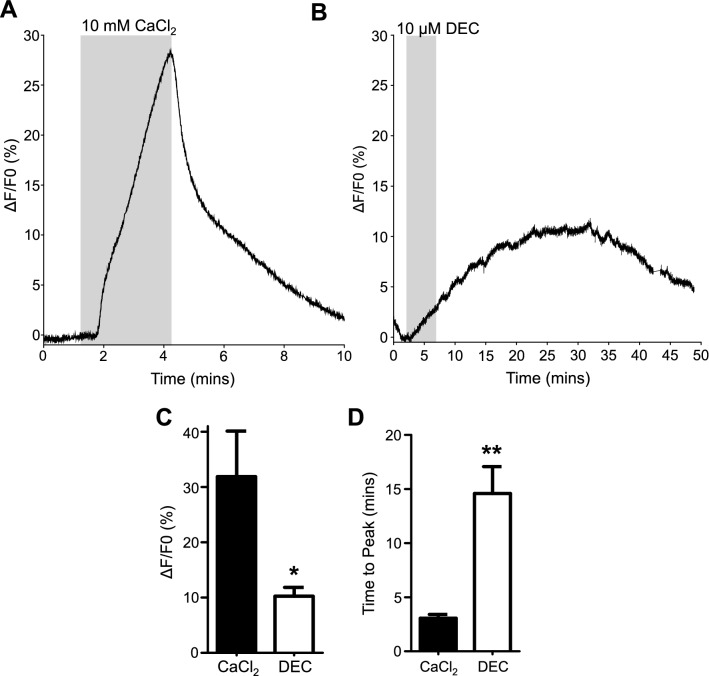
DEC stimulates Ca^2+^ signals in *Ascaris suum* intestines: (**A**) Representative Ca^2+^ signal in response to 10 mM CaCl_2_. Grey box indicates 10 mM CaCl_2_ application. (**B**) Representative Ca^2+^ response to 10 µM DEC. Grey box represents DEC application. (**C**) Total amplitudes of Ca^2+^ induced Fluo-3 fluorescence in response to 10 mM CaCl_2_ and 10 µM DEC. *significantly different to CaCl_2_ (CaCl_2_ vs DEC *P* < 0.0255, *t* = 2.827 *df* = 7, paired *t*-test) *n* = 8 intestines from 7 female worms. (**D**) Quantification of time for Ca^2+^ to reach maximum in response to 10 mM CaCl_2_ and 10 µM DEC. **significantly different to CaCl_2_ (CaCl_2_ vs DEC *P* < 0.0014, *t* = 5.122, *df* = 7 paired *t*-test) *n* = 8 intestines from 7 female worms. All values represented as means ± SEM.

We loaded the cells of the intestines of *A. suum* with the Ca^2+^ reporter Fluo-3AM (Methods). Increasing the Ca^2+^ concentration in the APF bathing solution from 1 to 10 mM provides a valuable control test of the physiological condition of the intestine preparation. In fresh preparations, it produced a rapid and smooth increase in the Fluo-3 fluorescence starting within 20 s of application. This Ca^2+^ induced rise in cytosolic calcium declined immediately after the bath CaCl_2_ concentration was returned to 1 mM. The time for the CaCl_2_ signal to reach the peak was relatively fast, taking a mean of 3 min, Fig. 4A and D. We point out that we observed no major fluctuations in the Ca^2+^ signal when the sample was constantly perfused long term with 1 mM CaCl_2_ APF; there was only small spontaneous fluctuations that had an average amplitude of 0.6% (Supplementary Fig. S2A and B).

Previous work on the muscle cells of the filarial nematode, *B. malayi*, showed that DEC opens their Transient Receptor Potential channels (TRPs), causing spastic paralysis^7^. We anticipated that DEC would also open TRP channels in the intestines of *Ascaris*. We bathed the intestines in 10 µM DEC for 5 min and observed that DEC produced a slow but uneven ‘sometimes step-like’ Ca^2+^ signal in the intestine as opposed to the smoother rise caused by the CaCl_2_ and was initiated within 30 s of the DEC application (Fig. 4B). The increase continued to rise after removal and washout of DEC before eventually declining; the Ca^2+^ concentration reached its peak at a mean of 14.5 min after DEC exposure (Fig. 4D). The peak amplitude of the DEC signal was significantly smaller (10%) and slower than that produced by the application of 10 mM CaCl_2_ (31%), Fig. 4C.

Although the DEC calcium signal was reversible on washing, we used histology to check that the observed calcium signal was not associated with DEC induced cellular damage of the enterocytes. We exposed different *Ascaris* body flaps (intestine and underlying muscle cells) from the same worms to 10 µM DEC for 5 min and then washed them in 1 mM CaCl_2_ APF for 10 min (average time to peak) or 60 min (average length of a recording). We then compared the enterocytes of the sections taken: before DEC treatment; 10 min after 5 min exposure to 10 µM DEC and; after the 60 min wash. We observed no evidence of DEC causing death or damage to the cell body of the enterocyte or their brush borders (Supplementary Fig. S3). Based on these observations, DEC generates a Ca^2+^ signal increase with unique characteristics that is reversible on washing and, not associated with histological damage.

### La^3+^ inhibits DEC cytosolic Ca^2+^ increases in normal extracellular Ca^2+^ and inhibits DEC cytosolic Ca^2+^ decreases in low extracellular Ca^2+^

**Figure 5:**
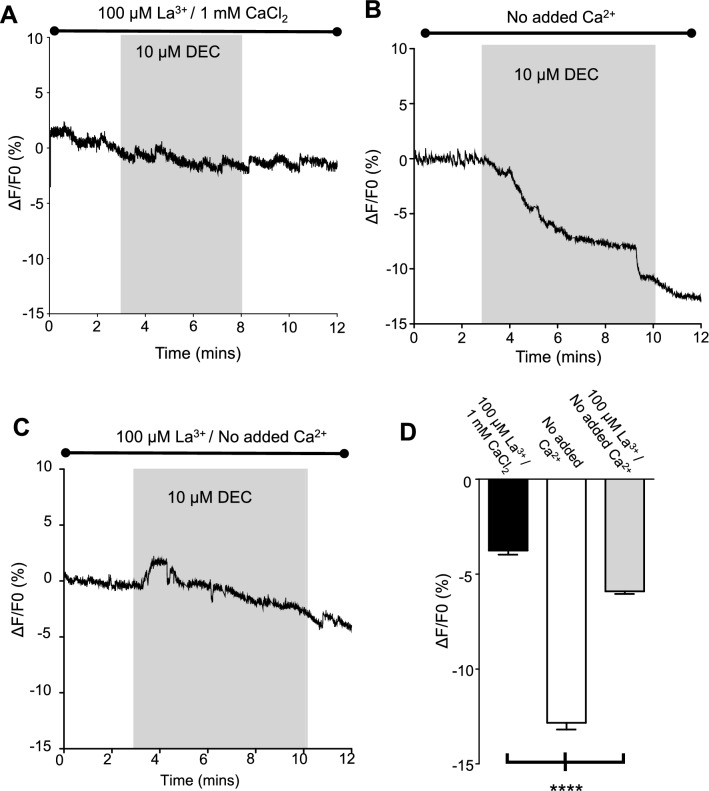
DEC Ca^2+^ signal changes are inhibited by La^3+^: (**A**) Representative trace of the inhibition of the effect of application of 10 µM DEC (grey box) by 100 µM La^3+^ in 1 mM extracellular Ca^2+^ APF. (**B**) Representative trace of the reduction of the Ca^2+^ signal produced by 10 µM DEC in no added Ca^2+^ APF. Grey box indicates 10 µM DEC application. (**C**) Representative trace of the inhibition by 100 µM La^3+^ of the reduction of the Ca^2+^ signal produced by 10 µM DEC in the no added Ca^2+^ APF. Grey box indicates 10 µM DEC application. (**D**) Histogram of the mean ± SEM reductions in Fluo-3 fluorescence in samples bathed in 100 µM LaCl_3_ and 1 mM CaCl_2_ bath solution (black bar), (*n* = 5 intestinal preparations from 5 individual female worms), samples bathed in no added CaCl_2_ APF (white bar) (*n* = 8 intestinal preparations from 6 individual female *Ascaris),* and samples bathed in 100 µM LaCl_3_ and no added Ca^2+^ bath solution (grey bar) (*n* = 10 intestinal preparations from 6 individual female worms) ***Significantly different to 100 µM LaCl_3_ and 1 mM CaCl_2_ bath solution (100 µM LaCl_3_ and 1 mM CaCl_2_ bath solution vs no added CaCl_2_ APF *P* = < 0.0001, *t* = 18.90, *df* = 748, unpaired *t*-test; 100 µM LaCl_3_ and 1 mM CaCl_2_ bath solution vs 100 µM LaCl_3_ and no added Ca^2+^ bath solution *P* = < 0.0001, *t* = 8.862, *df* = 748, unpaired *t*-test). All values represented as means ± SEM.

The TRP channels, GON-2/GTL-1 in the *C. elegans* intestine, have nearly identical pore domains^31–34^ and exhibit high Ca^2+^ selectivity, which is blocked rapidly by low extracellular La^3+^ concentrations (100 µM) preventing movement of calcium through the plasma membrane into the enterocytes^34–36^. We tested the effects of 100 µM La^3+^ on our DEC responses. Figure 5A shows a representative trace where 100 µM La^3+^ blocks the DEC increase in Ca^2+^ in normal extracellular Ca^2+^ APF. Interestingly, in low extracellular calcium (no La^3+^), DEC produces a reduction in the cytoplasmic calcium rather than an increase, due to leaching of intracellular calcium from the cell, Fig. 5B; and this leaching is inhibited by 100 µM La^3+^, Fig. 5C. These observations show that DEC can open Ca^2+^ permeable plasma membrane channels in the enterocytes that are blocked by La^3+^ without producing release of intracellular Ca^2+^. Figure 5D shows histograms of the significance and the means ± SEM changes in the Ca^2+^ signal produced in the different experiments, *n* = 5 preparations for each experiment. The entry of Ca^2+^ to, and loss of Ca^2+^ from, the cytosol through the plasma membrane channels that is inhibited by a low-concentrations of La^3+^, is consistent with DEC opening TRP channels. The effects of La^3+^ were readily washed off and control responses to our test 10 mM CaCl_2_ then returned (not shown).

Although La^3+^ is recognized for blocking TRP channels of the nematode intestine, it is possible that other plasma membrane channels permeable to calcium could be a target of DEC. The plasma membrane channels may include the voltage-activated calcium Ca_v_ channels, I_CRAC_ and other calcium entry pathways that are also blocked by La^3+^. We used 2-APB and SKF 96365 to explore the pharmacology of the DEC calcium signaling response and the role of TRP channels in the response.

### 2-APB inhibits DEC increases in cytosolic Ca^2+^

**Figure 6:**
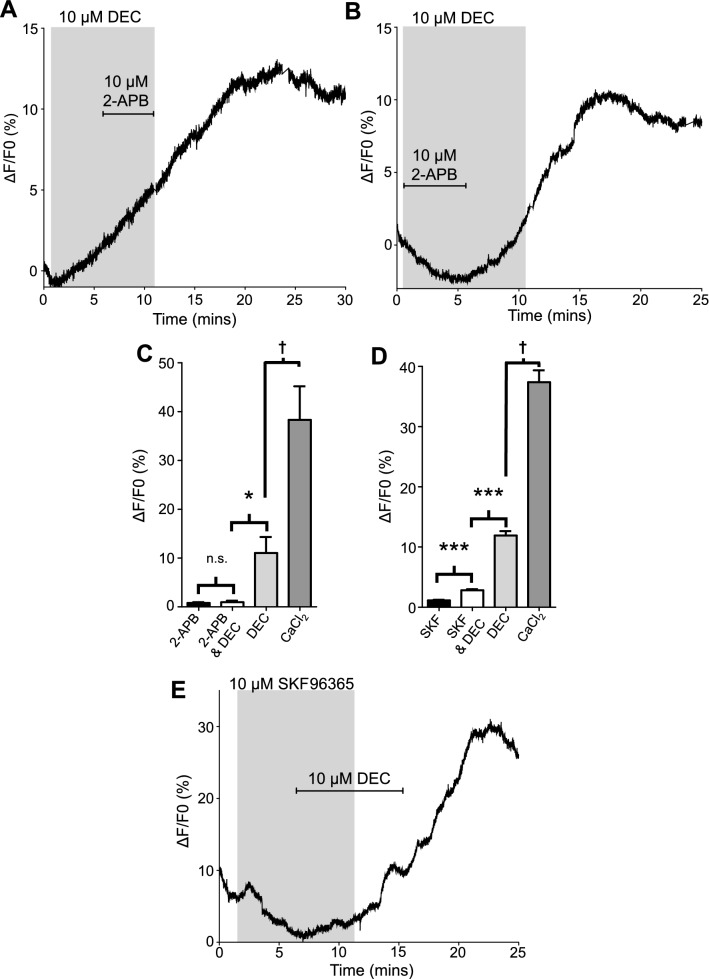
TRP channel antagonists 2-APB and SKF inhibit DEC mediated Ca^2+^ signals: (**A**) Representative trace in response to 10 µM 2-APB being applied after 10 µM DEC initiates a Ca^2+^ signal. Grey box represents DEC 10 µM application, bar represents 10 µM 2-APB. (**B**) Representative trace in response to 10 µM 2-APB being applied at the same time as 10 µM DEC. Grey box represents DEC 10 µM application, bar represents 10 µM 2-APB. (**C**) Total amplitudes of Fluo-3 fluorescence in response to 10 µM 2-APB (black bar), 10 µM 2-APB & 10 µM DEC (white bar), 10 µM DEC (light grey bar) and 10 mM CaCl_2_ (dark grey bar). N.S. not significantly different to 2-APB (2-APB vs 2-APB + DEC *P* = 0.6519, *t* = 0.4650, *df* = 10, unpaired *t-*test). 2-APB *n* = 6 intestines from 3 females; 2-APB + DEC *n* = 6 intestines from 4 females. *Significantly different to 2-APB + DEC (2-APB + DEC vs DEC *P* < 0.026 *t* = 3.102 *df* = 5, paired *t*-test). *n* = 6 intestines from 4 females. † Significantly different to DEC (DEC vs CaCl_2_ *P* < 0.0326, *t* = 2.931, *df* = 5, paired *t*-test). *n* = 6 intestines from 4 females. (**D**) Total maximal amplitudes of Fluo-3 fluorescence in response to 10 µM SKF96365 (black bar), 10 µM SKF96365 and 10 µM DEC (white bar), 10 µM DEC (light grey bar) and 10 mM CaCl_2_ (dark grey bar). ***Significantly different to SKF (SKF vs SKF + DEC *P* = < 0.0001, *t* = 7.38, *df* = 349, paired *t-*test). SKF96365 *n* = 7 intestines from 6 females; SKF96365 + DEC *n* = 7 intestines from 6 females. ***Significantly different to SKF96365 + DEC (SKF96365 + DEC vs DEC *P* < 0.0001 *t* = 14.74 *df* = 349, paired *t*-test). *n* = 7 intestines from 6 females. † Significantly different to DEC (DEC vs CaCl_2_ *P* < 0.0001, *t* = 14.82, *df* = 349, paired *t*-test). *n* = 7 intestines from 6 females E) Representative trace in response to 10 µM SKF96365 and 10 µM DEC. Grey box represents 10 µM SKF96365 application, bar represents 10 µM DEC. All values represented as means ± SEM.

We used the broad-spectrum TRP channel antagonist, 2-APB (2-aminoethoxydiphenyl borate) which at low concentrations (10 µM) also has stimulatory effects of Store Operated Calcium entry (SOC) mediated by I_CRAC_ channels^40^. We tested the effects of 10 µM 2-APB on DEC responses, Fig. 6. Application of 10 µM 2-APB alone had no effect on the Ca^2+^ signal (Fig. 6C; black bar). We found that 10 µM 2-APB did not inhibit the effect of 10 µM DEC if applied after the DEC: the Ca^2+^ response continued in the presence of 2-APB and after removal of DEC, Fig. 6A. However, if 2-APB was applied at the same time as DEC, it prevented any increase in the intracellular Ca^2+^ fluorescence (Fig. 6B and C, white bar) until the 2-APB was removed, which then generated an increase in fluorescence that continued after DEC was removed (Fig. 6B and C, light grey bar). The lack of 2-APB inhibition when applied after the DEC may be explained by calcium-induced calcium release into the cytoplasm once there is an initial rise in cytosolic calcium.

We checked the response to 10 mM CaCl_2_ after 2-APB and DEC applications to ensure that the preparation was still responding normally and saw the usual ~ 30% increase in the fluorescent signal (Fig. 6C, dark grey bar). Thus, 2-APB does not irreversibly inhibit the Ca^2+^ signaling machinery. The histogram shown in Fig. 6C summarizes the mean % changes in Ca^2+^ fluorescence changes from 6 similar experiments. There was no significant increase in Ca^2+^ when 2-APB was co-applied with DEC (Fig. 6C, compare black and white bars), but when 2-APB was removed in the presence of DEC, we observed a significant increase in the Ca^*2*+^ signal with its characteristic phenotype and similar peak (Fig. 6B and C, compare white bar with the light grey bar). Although La^3+^ can block other channels, including voltage-gated calcium channels, Ca_v_s, and other cation permeable plasma channels^37^, 2-APB has no inhibitory effect on voltage-gated calcium channels^38,39^ and at low (10 µM) concentrations can stimulate SOC extracellular Ca^2+^ entry pathways mediated by I_CRAC_ (Orai) channels^40^. The inhibitory effects of La^3+^ and 2-APB together can be explained by the effects of DEC opening TRP channels in the plasma membrane to promote Ca^2+^ entry into the intestinal cells via TRP channels.

### SKF 96365 inhibits DEC signaling

We have pointed out that the broad-spectrum TRP inhibitor, 2-APB, may have off target effects including opening of I_CRAC_ SOCs^38–40^ and that La^3+^ blocks calcium entry pathways including: TRPs and voltage-activated calcium Ca_v_ channels^37^. We wanted to determine if our observed DEC signal included a role for a more specific TRP channel subtype. We exposed intestinal tissues to the TRPC selective antagonist SKF 96365^40,41^. We have previously demonstrated that 10 µM SKF 96365 is able to inhibit DEC mediated inward currents in *Brugia* muscles^7^. We exposed intestinal tissues to 10 µM SKF 96365 for 5 min and observed no changes in the Ca^2+^ amplitude (Fig. 6E and D, black bar). Upon application of 10 µM DEC in addition to SKF 96,365, we observed that the DEC mediated signal was inhibited, with the average amplitude being reduced to 3% (Fig. 6E and D, white bar). The effect of SKF 96365 was washed off, reversing the inhibition, and allowing DEC to stimulate a Ca^2+^ signal with an average amplitude of 11% (Fig. 6E and D, light grey bar) and the response to the 10 mM Ca^2+^ test to be unaltered (Fig. 6D, dark grey bar). Although SKF 96365 has TRPC selective effects it may have off-target effects that include: SOCs^40^, voltage-activated calcium channels (T-type Ca_V_ channels^41^) and, K^+^ channels^39,42^. However, of all antagonists we tested, there is one target in common, the TRP channels. Our results here indicate that DEC gives rise to the entry of calcium into the intestine by opening plasma membrane TRP channels as we have observed in *Brugia* muscle. The continued increase in Ca^2+^ fluorescence after removal of DEC may relate to a slow wash-off effect of DEC and downstream secondary Ca^2+^ induced Ca^2+^ release from intracellular stores^35^.

### Prolonged DEC exposure does not change TRP mRNA levels in the intestine or muscle

Maintained applications of diethylcarbamazine to adult *B. malayi* produces a temporary spastic paralysis that lasts for less than 4 h that is followed by desensitization and recovery^7^. We were interested in determining if there was accommodation to DEC in *A. suum* mediated by reducing TRP channel message during a 4-h exposure. We treated isolated intestine flaps and muscle flaps with 10 and 100 µM DEC for 4 h. Again, we used qPCR to measure relative mRNA levels of the six TRP channels in the treated flaps and compared them to untreated controls that were bathed in RPMI. We observed no significant changes in message levels for any TRP channels in either the intestines or muscle cells samples treated with either 10 µM or 100 µM DEC when using a one-way ANOVA with a post-hoc Tukey test (Supplementary Fig. S5A and B). These observations show that the level of TRP channel transcript expression in the intestines and muscle cells of *A. suum* is not sensitive to DEC over this timescale.

## Discussion

The nematode intestine carries out essential functions for survival, including: (1) digestion and nutrient absorption^10,12^; (2) pH regulation via apical membranes V-type ATPases^18^; (3) storage of lipids^43^; (4) innate immunity^14,44^; (5) secretion of bactericidal peptides^15^; (6) drug metabolism with P450 cytochromes and UDP transferases^17^ and; (7) excretion by organic-anion-transporter (OAT-1), P-glycoprotein- and MDR-transporters into the lumen of the intestine^14,18,45^. Damage to the cells of the intestine will adversely affect these vital functions.

The intestine cells are columnar cells, electrically coupled to their neighbors, and polarized with the apical region bearing microvilli and the basolateral region facing the somatic body muscle cells. The digestion of food involves the secretion of proteases and lipases and the absorption of nutrients including glucose and lipids. It is facilitated by a range of selective transporters^46^. The processes of absorption of nutrients and secretion of proteases also require significant endocytosis and exocytosis utilizing different types of vacuoles and vesicles that are present in the intestinal cells; these vesicles are moved within the cell on an elaborate network of microtubules that includes a terminal web under the apical microvilli^11^. Absorption of Ca^2+^ and Mg^2+^ is mediated by TRPM-like ion-channels (GON-2)^35,36^.

**Figure 7:**
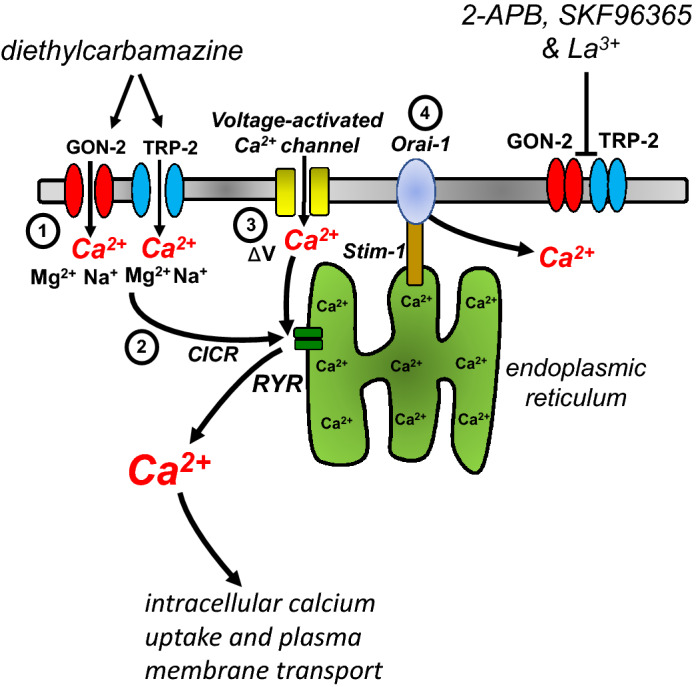
Summary diagram of proposed DEC stimulation of Ca^2+^ entry into intestinal cells via TRP channels: Diagram representing the mode of action for DEC in the intestine of *ascaris suum*. (1). DEC activates the TRPM channel GON-2 (red) and TRPC channel TRP-2 (blue), to promote extracellular Ca^2+^ entry. Cation entry can lead to two possible pathways (2) Ca^2+^ induced Ca^2+^ release (CICR) leading to intracellular storage release via RYR receptors (green). (3) activation of Ca^2+^ channels (yellow) on the membrane by changes in membrane potential (ΔV) increasing extracellular Ca^2+^ entry. (4) Emptying of the calcium stores in the endoplasmic reticulum activates store-operated entry mediated by STIM1 and Orai-1. 2-APB, SKF96365 and La^3+^ can inhibit TRP channels preventing Ca^2+^ entry and subsequent Ca^2+^ increases. Cytosolic calcium is subsequently taken up in intracellular organelles or removed by exchangers and ATPases.

The intestine of parasitic nematodes is increasingly being recognized as a major site of action of a number of anthelmintic drugs in addition to the neuromuscular system^47^. These anthelmintics now include:

1. *Diethylcarbamazine (DEC):* The disturbance in the Ca^2+^ homeostasis of the intestine by an action of DEC on TRP channels can disrupt vital functions of the enterocytes and will contribute to the anthelmintic action of DEC against ascariasis. DEC activates TRP-2, GON-2 & CED-11 channels that produce an inward sarcolemma current in *Brugia malayi* muscle cells where 2-APB and SKF 96365 are also effective as DEC antagonists^7^. The presence of TRP channel message in *Ascaris* intestine that is similar to that of *B. malayi*, Fig. 1 suggests that DEC will have the same effects on the *A. suum* intestine. The actions of La^3+^ and 2-APB taken together on the intestine inhibiting the actions of DEC are explained by DEC also opening TRP channels in the intestine of *A. suum.* The effect of SKF 96365 also supports a role for TRP-2 channels.
  Fig. 7 shows a summary model of the action of DEC on the *Ascaris* intestine mediated by activating TRP-2, and GON-2 TRP channels leading to the entry of Ca^2+^ into the intestinal cells. We have included here STIM-1 and ORAI-1 (Supplementary Fig. S4 and Table S3) that is involved in store-operated calcium entry and ryanodine receptors that are involved in calcium-induced calcium release, although details of their role in the DEC response requires further study. Message for GON-2 and TRP-2 channels are present in the *A. suum* intestine as they are in *B. malayi* muscle cells^7^. DEC also produces excitability and depolarization in *A. suum* muscle cells^48^ showing that DEC has actions on both muscle cells and intestine cells of related clade III nematode parasites, Fig. 1. We point out that we also found *Asu-ced-11, ocr-1, osm-9* and *Asu-trpa-1* message in the intestine and effects of DEC on these TRP channels were not excluded by our observations. However, the relatively high expression levels of *Asu-gon-2* and the presence of *Asu-trp-2* in the intestine compared with the muscle and their role in mediating DEC effects in *B. malayi* muscle favors GON-2 and TRP-2 as targets of the action for DEC in the intestine. Also, the TRP channel antagonists, 2-APB and SKF96365, inhibit the responses of the intestine to DEC as it does on *B. malayi* muscle, suggesting a similar mode of action. Further support for the action of DEC on TRP channels comes from the effects of La^3+^ that inhibits TRP channels, and responses to DEC^35^ and; interestingly, in the calcium-free conditions, DEC produces a fall in calcium, suggesting that cytosolic calcium is leached through the DEC activated TRP channels.
2. *Benzimidazoles:* The intestine of nematodes is sensitive to the actions of the benzimidazole anthelmintics, mebendazole, and albendazole^19,49,50^. These drugs bind selectively to the *β*-tubulin of nematodes, disrupt microtubules, and the movement of digestive vacuoles and lysosomes, leading to autolysis of the intestinal cells^19,49^.
3. *Levamisole:* We have reported that the intestine expresses message for different acetylcholine gated ion channels subunits, including UNC-38, UNC-29, UNC-63, and ACR-8^21^. The application of the anthelmintic levamisole opens these nicotinic acetylcholine channels that produce a clear and characteristic calcium signal. The function of these channels in the intestine cells may be paracrine, to communicate locally with adjacent intestine cells and underlying muscle cells, because the cells are not electrically excitable like muscle cells or nerve cells.
4. The *Bacillus thuringiensis toxin:* Cry5B is a potent pore-forming toxin that can target multiple different species of gastrointestinal parasites including *Ascaris suum*^26–28^. Cry5B appears to have two modes of action: (1) binding selectively to the CDH-8 cadherin present on the surface of the cell membrane of *C. elegans* intestines, leading to oligomerization of the toxin and possible internalization^25^ and; (2) interaction with glycolipids produced by BRE-5, a glycosyltransferase that catalyzes the addition of monosaccharides onto glycolipids, leading to Cry5B insertion into the membrane forming the pore^22,23^. Interestingly, *C. elegans* that are resistant to the anthelmintic levamisole, show increased hyper-susceptibility to Cry5B and vice versa with Cry5B resistant strains showing increased sensitivity to levamisole, resulting in a synergistic relationship between two anthelmintics^23^. Here, we have observed that DEC has a direct effect on the intestine, in addition to the known effects of levamisole^21^. In addition to levamisole, the two other cholinergic agonist anthelmintics, pyrantel and tribendimidine also have strong synergistic effects with Cry5B in *C. elegans*^23^ that is also seen in the hookworm, *Ancylostoma ceylanicum*^24^. The observations that we describe here suggest a mechanism by which DEC will add to, and potentiate, the effects of albendazole, levamisole, ivermectin, and Cry5B in combinations treatments for STH infections.

We have pointed out above that the nematode parasite intestine is a site of action of DEC, the benzimidazoles, cholinergic anthelmintics like levamisole, and Cry5B. These anthelmintic drugs disrupt the normal Ca^2+^ homeostasis and vital functions and suggest that combinations of these anthelmintics will have additive or synergistic effects. Adverse effects on the intestine will potentiate the effects of other anthelmintics that are metabolized or excreted by the intestine. One example is the macrocyclic lactone, ivermectin, which acts as positive allosteric modulators of the Glutamate-gated Chloride channel. P-glycoproteins have been suggested as candidate genes for ivermectin resistance by acting as efflux pumps. The P-glycoprotein gene *pgp-9* has been repeatedly associated with macrocyclic lactone resistance in different nematode parasites^51,52^. Although P-glycoproteins are found in other nematode tissues, they are most strongly expressed in the intestine of the parasites^45,53^. The combination of albendazole with ivermectin or diethylcarbamazine is anticipated to enhance the efficacy of the treatment of STHs compared to albendazole alone, ivermectin alone, or diethylcarbamazine alone. This has, in fact, been seen in the study of the effects of albendazole, ivermectin, and diethylcarbamazine alone and combinations against *ascariasis* and *trichuriasis*^54^. The parasite intestine offers a common target where anthelmintics can have synergistic effects.

## Materials and methods

### Collection and maintenance of *A. suum* worms

Adult female *A. suum* worms were collected from the JBS Swift and Co. pork processing plant at Marshalltown, Iowa. Worms were maintained in *Ascaris* Ringers Solution (ARS: 13 mM NaCl, 9 mM CaCl_2_, 7 mM MgCl_2_, 12 mM C_4_H_11_NO_3_, Tris, 99 mM NaC_2_H_3_O_2_, 19 mM KCl and 5 mM glucose pH 7.8) at 32 °C for 24 h to allow for acclimatization before use in experiments. The solution was changed daily, and worms were used for a maximum of three days for experiments. All the worms were examined at the start of each day and excluded if they were damaged or immotile.

### *A. suum* cDNA synthesis and RT-PCR detection of TRP channels

Dissection of muscle bags and intestinal tissue was conducted on adult *A. suum* females as previously described, McHugh et al., 2020. Subsequently, muscle bags and intestinal tissue were homogenized separately in 1 ml of Trizol reagent using a mortar and pestle, followed by total RNA extraction according to the Trizol Reagent protocol (Life Technologies, USA). One microgram (1 µg) of total RNA from each tissue was used to generate cDNA by reverse transcription (RT) using SuperScript IV VILO™ Master Mix (Life Technologies, USA) following the manufacturer's protocol. PCR was conducted to detect the presence of *Asu-gon-2*, *Asu-trp-2*, *Asu-ced-11*, *Asu-ocr-1*, *Asu-osm-9*, and *Asu-trpa-1* using primers that were designed for targeting encoding regions of each gene (Supplementary Table S1)*. Asu-gapdh* was used as a reference gene. Negative controls included enzyme, water, and both forward and reverse primers for the target with no cDNA template. The cycling conditions for PCR were an initial denaturation for 2 min at 95 °C, followed by 35 cycles of 95 °C for 30 s, 58 °C for 35 s, 72 °C for 45 s, and a final extension at 72 °C for 10 min using GoTaq® G2 Hot Start Green Master Mix (Promega, USA). The PCR products of each gene were then separated on an individual 1% Agarose containing SYBR® Safe DNA Gel Stain (ThermoFisher Scientific), for 45 min at 100 V, followed by visualization under UV light to confirm the presence of the genes. All photographs were acquired using Visionworks™ software (Analytik Jena) with an exposure setting of 3 s per 1 frame. Original gel pictures are presented in Supplementary Fig. S1.

### Analysis of mRNA levels by quantitative real-time PCR

To quantify the relative mRNA transcript levels of each identified TRP channel in the intestine and muscle bags, cDNA was synthesized from 1 µg of RNA from each adult female *A. suum* as described above. Target genes with fragments ranging from 150 to 200 bp were amplified by qPCR from each cDNA sample in triplicate. The same procedure was used for the reference gene *Asu-gapdh*. All primers for qPCR are presented in Supplementary Table S2. The quantitative PCR reaction mixture consisted of 1 μl of cDNA template, 1 μl of forward and reverse primer mix, and 10 μl of PowerUp™ SYBR™ Green Master Mix (Applied Biosystems, ThermoFisher, USA), with the final volume made up to 20 μl with Nuclease-free water. The cycling conditions included an initial denaturation for 10 s at 95 °C, 40 cycles of 95 °C for 15 s and 58 °C for 30 s followed by a final melting curve step. Cycling was performed using a QuantStudio™ 3–96 well 0.1 mL Block Real time PCR Detection system (ThermoFisher, USA), and transcript quantities were derived by the system software, using the generated standard curves. mRNA expression levels for each subunit (*Asu-gon-2, Asu-trp-2, Asu-ced-11, Asu-ocr-1, Asu-osm-9, and Asu-trpa-1*) were estimated relative to the reference gene (*Asu-gapdh*) using the Pfaffl Method. The qPCR experiments were repeated 3 times for each gene and for each DEC treatment (all subunit mRNA quantifications were performed in triplicate for each worm's muscle bag sample and intestinal tissue sample: 15 biological replicates each with three technical replicates).

### Dendrogram construction

All protein sequences used for the construction of the dendrogram were acquired either from Wormbase Parasite, The European Nucleotide Archive (ENA) or UNIPROT repositories. Accession number links to sequences used for each gene of each organism can be found in Supplementary Table 3. All protein alignments were done using MAFFT (http://mafft.cbrc.jp/alignment/software/)^55^. Amino-acid similarity and identity was analyzed using MAFFT alignments. Percent identity was the percent ratio of identical amino-acids and the total number of residues in the longest sequence. Similarly, percent similarity was determined by dividing the number of identical and similar amino-acids by the total number of residues in the longest sequence (× 100). Phylogenetic analysis was done using the software MEGA X software^56^ using the maximum likelihood method based on Le and Gascuel model^57^.

### Preparation and loading Fluo-3AM

A 2 cm section of the intestine was removed from the body piece using fine forceps and cut open. The intestinal flap was placed onto a coverslip (24 × 50 mm) and pinned using a slice anchor (26 × 1 mm × 1.5 mm grid, Warner Instruments, Hamden, CT), immersed in *Ascaris* Perienteric Fluid APF (23 mM NaCl, 110 mM NaAc, 24 mM KCl, 1 mM CaCl_2_, 5 mM MgCl_2_, 5 mM HEPES, 11 mM D-glucose) in a laminar flow chamber (Warner RC26G, Warner Instruments, Hamden, CT). Fluo-3AM loading was achieved by incubating the intestine in APF solution with no added CaCl_2_ (< 100 µM Ca^2+^) containing 5 µM Fluo-3AM and 10% Pluronic F-127 (10% v/v) for one hour with the chamber connected to a Dual Automatic Temperature Controller (Warner Instruments, Hamden, CT) maintained at 34–36 °C. After incubation, the Fluo-3AM solution was discarded, and the sample was incubated in APF containing 1 mM CaCl_2_ for an additional 15 min at 34–36 °C to promote Ca^2+^ loading. Intestinal tissues were then continuously perfused with APF containing 1 mM CaCl_2_ and exposed to either 10 µM diethylcarbamazine (DEC), 10 µM 2-APB, 10 µM DEC and 2-APB, 10 µM SKF96365, 10 µM SKF96365 and DEC or 10 mM CaCl_2,_ which was used as a positive control. For no added (low calcium) Ca^2+^ experiments, 1 mM CaCl_2_ APF was exchanged for no added CaCl_2_ APF (measured < 100 µM Ca^2+^) after 10 min incubation and the samples were incubated for an additional 5 min before the beginning of each experiment at 34–36 °C. During these recordings, preparations were continually washed in solutions with no added CaCl_2_. For recordings with La^3+^, intestinal preparations were incubated in 1 mM CaCl_2_ APF containing 100 µM LaCl_3_ to inhibit TRP channels for 10 min. These intestine preparations were the incubated in no added CaCl_2_ and 100 µM LaCl_3_ for additional 5 min at 34–36 °C. For all experiments, samples were exposed to solutions containing no added CaCl_2_ and 100 µM LaCl_3_. All incubations were done in the absence of light to prevent degradation of the fluorescent dye.

All solutions were delivered to the chamber under gravity feed through solenoid valves controlled using a VC-6 six-channel Valve Controller (Warner Instruments, Hamden, CT) through an inline heater set at 37 °C (Warner Instruments, Hamden, CT) at a rate of 1.5 mL/min. At the start of all experiments, intestinal preparations were left under blue light for a minimum of 3 min to promote settling and equilibration of the fluorescent signal and monitor for any spontaneous Ca^2+^ signaling before application of any compound.

### Measurement of Ca^2+^ fluorescence

All recordings were performed on an Eclipse TE3000 microscope (20X/0.45 Nikon PlanFluor objective), fitted with a Photometrics Retiga R1 Camera (Surrey, BC, Canada). Light control was achieved using a Lambda 10–2 two-filter wheel system with a shutter controller (Lambda Instruments, Switzerland). Filter wheel one was set on a green filter between the microscope and camera. Filter wheel two was set on the blue filter between a Lambda LS Xenon bulb light box, which delivered light via a fiber optic cable to the microscope (Lambada Instruments, Switzerland). Blue light emission was controlled by using a shutter. Minimal illumination exposure was used to prevent photobleaching.

All Ca^2+^ signal recordings were acquired and analyzed using MetaFluor 7.10.2 (MDS Analytical Technologies, Sunnyvale, CA) with exposure settings at 150 ms with 2 × binning. Maximal percent Ca^2+^ signal amplitudes (ΔF) were calculated using the equation F1–F0/F0 × 100, where F1 is the fluorescent value and F0 is the baseline value. All F0 values were determined as being the value when any stimulus was applied to the sample for all traces analyzed.

*Details of numbers of regions and numbers of preparations used for measurements of the* Ca^2+^ *signals.*

Calcium signals from each intestine were collected from 50 square 50 µm × 50 µm areas across the intestine covering a total area of 125,000 µM^2^ that included 800–1000 individual enterocytes. The average fluorescence amplitude was calculated for each intestinal exposure of all 50 regions. For the DEC experiments, intestines were exposed to 10 µM DEC for 5 min followed by 10 mM CaCl_2_ as the positive control. For the 2-APB experiments intestines were exposed to 10 µM 2-APB for 5 min followed by 10 mM CaCl_2_. For the study of the effects of 2-APB on the DEC responses, preparations were exposed to 10 µM 2-APB and 10 µM DEC at the same time for 5 min before 2-APB was removed and DEC was left for an additional 5 min. 10 mM CaCl_2_ was then used as a positive control. For SKF 96,365 experiments, 10 µM SKF96365 was applied for 5 min followed by applying of 10 µM SKF96365 + 10 µM DEC for 5 min. SKF96365 was removed, and the DEC was left on for an additional 5 min. 10 mM CaCl_2_ was then used as a positive control. Finally, for the no added Ca^2+^ and La^3+^ experiments 10 µM DEC was applied for 5 min before subjected to 10 mM CaCl_2_.

### Histology

Sections of *Ascaris* body sections were cut along the lateral line and opened, exposing the intestine. Sections were then incubated in 1 mM CaCl_2_ APF containing 10 µM DEC for 5 min and then transferred to 1 mM CaCl_2_ APF for 10 min, average time for Ca^2+^ signal to reach peak (15 min total) or 55 min, the average length of recording (60 min total). Control samples were exposed to 1 mM CaCl_2_ APF for 3 min.

Following treatment, the segments of adult *Ascaris* worms were preserved in 10% buffered neutral formalin. Preserved specimens were placed in histology cassettes for paraffin embedding and sectioning. Hematoxylin and eosin staining was performed on 5 µm slides as described previously^58^. Histological sections were reviewed by an ACVM board-certified parasitologist (Brewer). Sections were viewed and images were captured on an Olympus BX60 microscope fitted with an Olympus DP70 camera (Olympus, Tokyo, Japan). Image captures were achieved using Olympus DP controller software (Olympus, Tokyo, Japan) under 20 × magnification and exposure settings of 1/350 s per frame.

### Statistical analysis

Statistical analysis of all data was done using GraphPad Prism 5.0 (Graphpad Software, Inc., La Jolla, CA, USA). To ensure reproducibility, we repeated our experiments: the numbers of female worms, intestine preparations, the concentrations, and durations of applications of 2-APB, DEC, La^3+^ are provided in the legends of the figures. Analysis of Ca^2+^ amplitudes and time to peak were done using either unpaired or paired student *t*-tests *P* < *0.05*. For qPCR experiments one-way analysis of variance (ANOVA), with a post-hoc Tukey test *P* < *0.05* was used. The data are presented as relative expression levels in mean ± SEM for each TRP channel subunit relative to *Asu-gapdh* in muscle bags and intestinal tissue. Comparisons were made using Tukey's post hoc test.

### Chemicals

Source of chemicals: 2-APB and SKF96365 were procured from Tocris; Sigma Aldrich supplied all other chemicals.

## Supplementary Information

Supplementary Information 1.

Supplementary Information 2.

Supplementary Information 3.

Supplementary Information 4.

Supplementary Information 5.

Supplementary Information 6.

Supplementary Information 7.

## Data Availability

The datasets analyzed during the current study are available in Wormbase Parasite, The European Nucleotide Archive (ENA) and UNIPROT repositories, https://parasite.wormbase.org/index.html, www.elixir-europe.org/platforms/data/core-data-resources, www.uniprot.org.
