# Supplementary material for: Diethylcarbamazine, TRP channels and Ca2+ signaling in cells of the Ascaris intestine
Source: Sci Rep. 2022 Dec 9;12:21317. doi: 10.1038/s41598-022-25648-7 (PMC9734116; doi:10.1038/s41598-022-25648-7)
Supplement: Supplementary file 1 — Supplementary Information 1. [file 41598_2022_25648_MOESM1_ESM.pptx]

## Slide 1
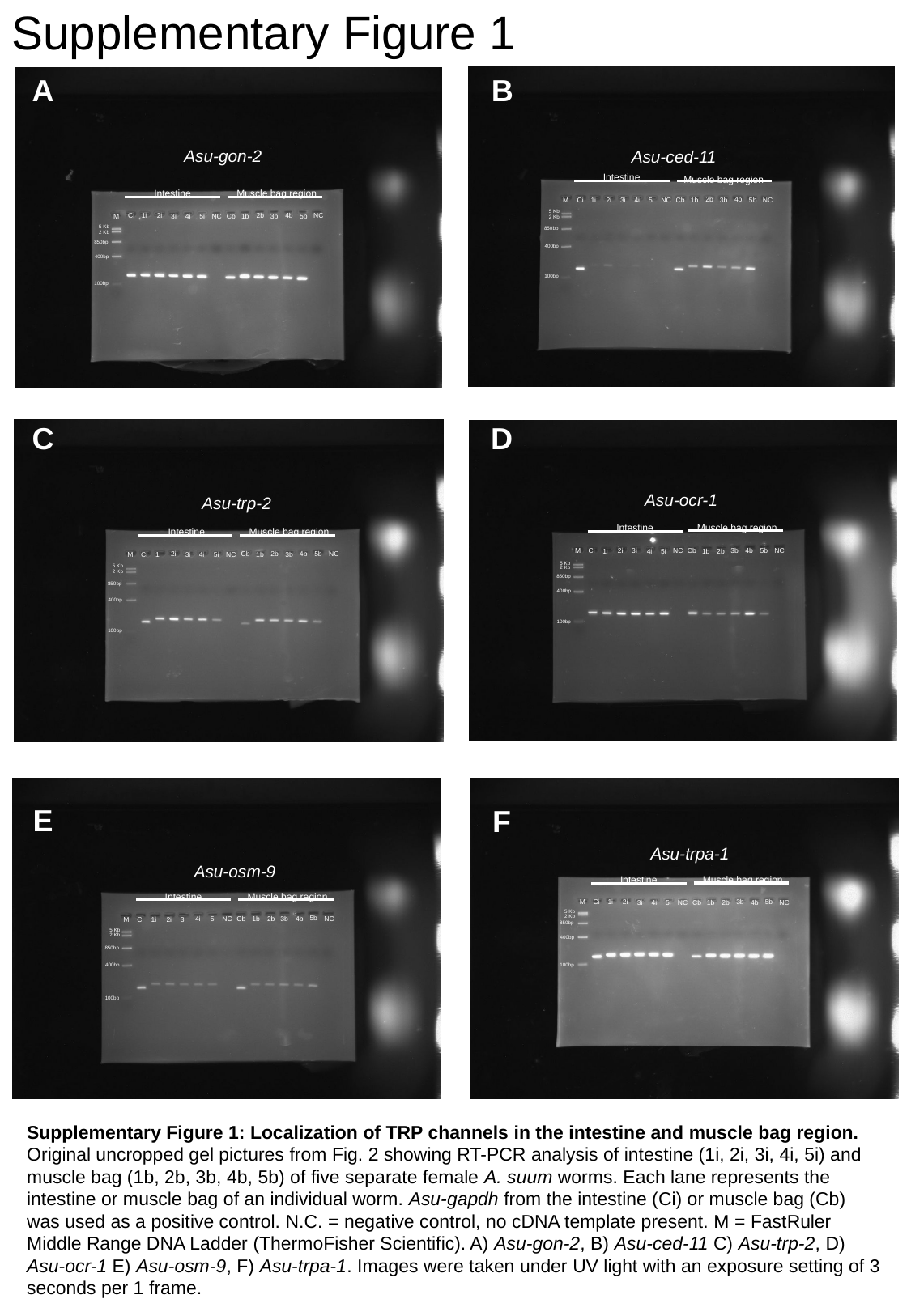

Supplementary Figure 1
A
B
Asu-gon-2
Asu-ced-11
Intestine
Muscle bag region
Intestine
Muscle bag region
2b
4b
NC
Ci
1i
2i
5b
1b
3b
3i
4i
5i
Cb
NC
M
2b
4b
NC
Ci
1i
2i
5b
1b
3b
3i
4i
5i
Cb
NC
M
5 Kb
2 Kb
5 Kb
850bp
2 Kb
850bp
400bp
400bp
100bp
100bp
D
C
Asu-ocr-1
Asu-trp-2
Intestine
Muscle bag region
Intestine
Muscle bag region
NC
4b
Ci
2i
5b
3b
3i
Cb
M
NC
2b
1i
1b
4i
5i
Cb
2b
4b
NC
2i
5b
1b
3b
M
3i
4i
5i
NC
Ci
1i
5 Kb
5 Kb
2 Kb
2 Kb
850bp
850bp
400bp
400bp
100bp
100bp
E
F
Asu-trpa-1
Asu-osm-9
Intestine
Muscle bag region
Intestine
Muscle bag region
M
Ci
1i
2i
5b
3b
3i
4i
5i
Cb
NC
2b
4b
NC
1b
5b
3b
Cb
2b
4b
NC
1b
4i
5i
NC
3i
Ci
1i
2i
M
5 Kb
2 Kb
850bp
5 Kb
2 Kb
400bp
850bp
100bp
400bp
100bp
Supplementary Figure 1: Localization of TRP channels in the intestine and muscle bag region. Original uncropped gel pictures from Fig. 2 showing RT-PCR analysis of intestine (1i, 2i, 3i, 4i, 5i) and muscle bag (1b, 2b, 3b, 4b, 5b) of five separate female A. suum worms. Each lane represents the intestine or muscle bag of an individual worm. Asu-gapdh from the intestine (Ci) or muscle bag (Cb) was used as a positive control. N.C. = negative control, no cDNA template present. M = FastRuler Middle Range DNA Ladder (ThermoFisher Scientific). A) Asu-gon-2, B) Asu-ced-11 C) Asu-trp-2, D) Asu-ocr-1 E) Asu-osm-9, F) Asu-trpa-1. Images were taken under UV light with an exposure setting of 3 seconds per 1 frame.
