# Supplementary material for: Diethylcarbamazine, TRP channels and Ca2+ signaling in cells of the Ascaris intestine
Source: Sci Rep. 2022 Dec 9;12:21317. doi: 10.1038/s41598-022-25648-7 (PMC9734116; doi:10.1038/s41598-022-25648-7)
Supplement: Supplementary file 2 — Supplementary Information 2. [file 41598_2022_25648_MOESM2_ESM.pptx]

## Slide 1
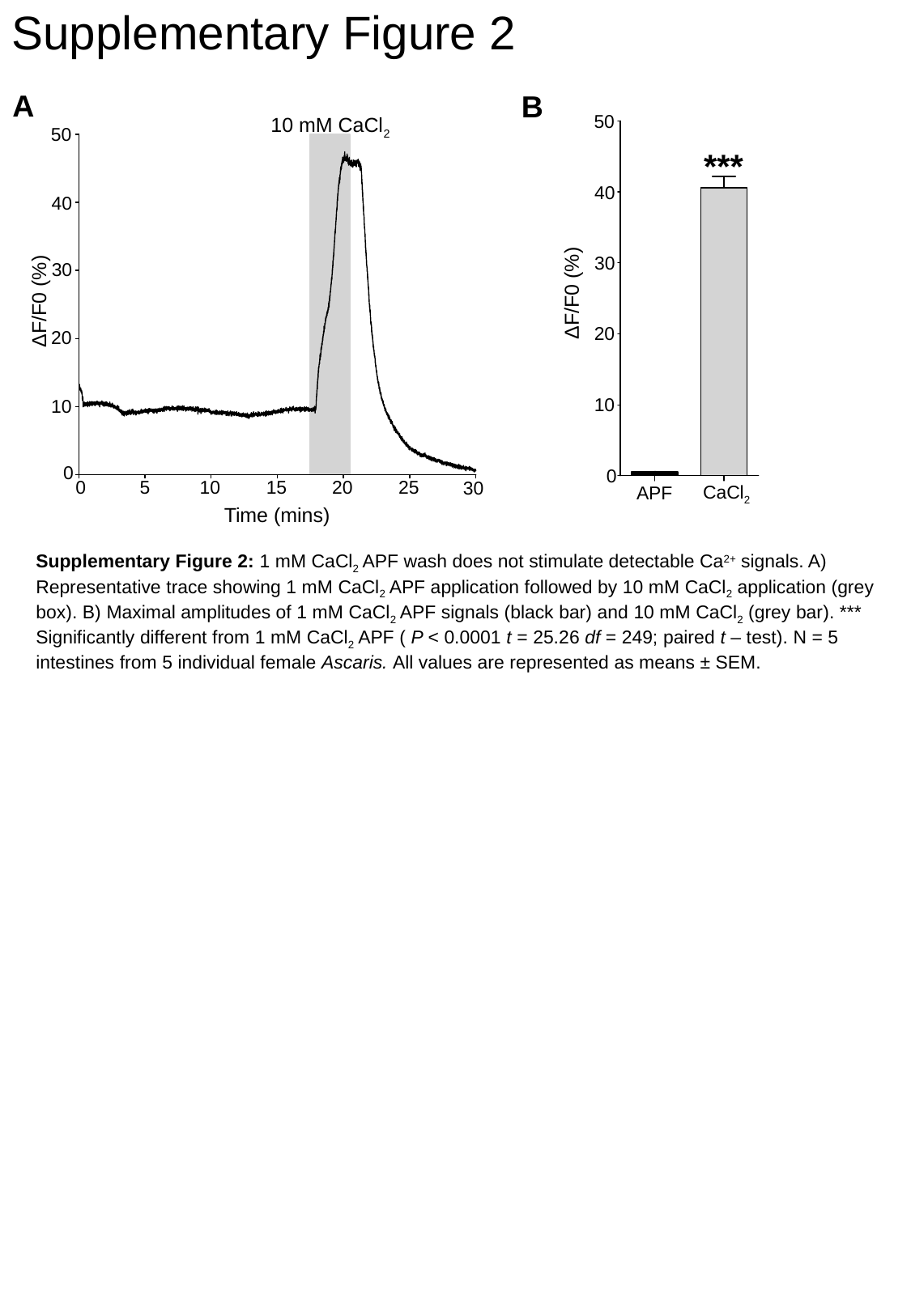

Supplementary Figure 2
A
B
50
10 mM CaCl2
50
40
30
ΔF/F0 (%)
20
10
0
0
15
5
10
20
25
30
Time (mins)
***
40
30
ΔF/F0 (%)
20
10
0
CaCl2
APF
Supplementary Figure 2: 1 mM CaCl2 APF wash does not stimulate detectable Ca2+ signals. A) Representative trace showing 1 mM CaCl2 APF application followed by 10 mM CaCl2 application (grey box). B) Maximal amplitudes of 1 mM CaCl2 APF signals (black bar) and 10 mM CaCl2 (grey bar). *** Significantly different from 1 mM CaCl2 APF ( P < 0.0001 t = 25.26 df = 249; paired t – test). N = 5 intestines from 5 individual female Ascaris. All values are represented as means ± SEM.
