# Supplementary material for: Diethylcarbamazine, TRP channels and Ca2+ signaling in cells of the Ascaris intestine
Source: Sci Rep. 2022 Dec 9;12:21317. doi: 10.1038/s41598-022-25648-7 (PMC9734116; doi:10.1038/s41598-022-25648-7)
Supplement: Supplementary file 3 — Supplementary Information 3. [file 41598_2022_25648_MOESM3_ESM.pptx]

## Slide 1
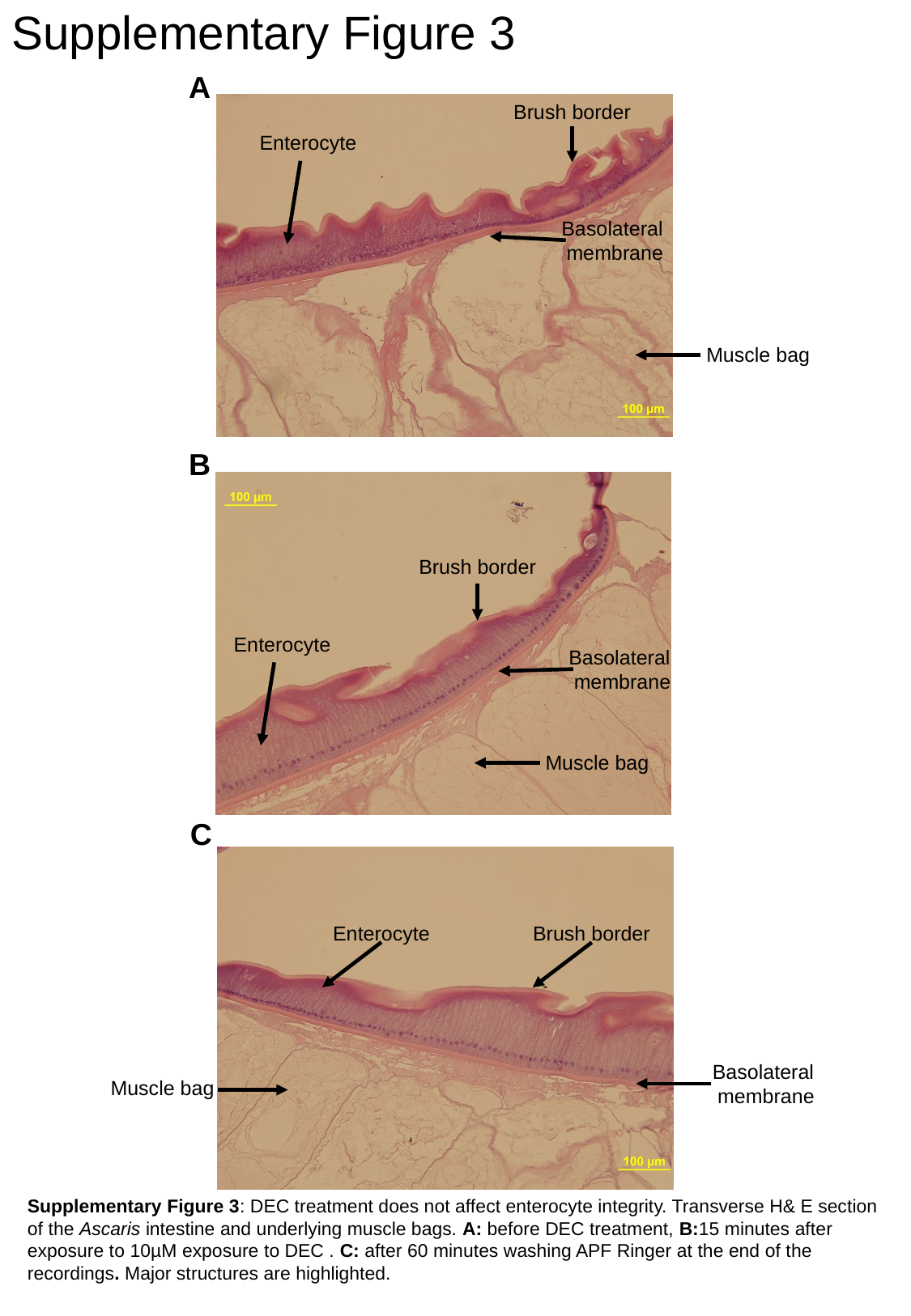

Supplementary Figure 3
A
Brush border
Enterocyte
Basolateral
membrane
Muscle bag
B
Brush border
Enterocyte
Basolateral
membrane
Muscle bag
C
Enterocyte
Brush border
Basolateral
membrane
Muscle bag
Supplementary Figure 3: DEC treatment does not affect enterocyte integrity. Transverse H& E section of the Ascaris intestine and underlying muscle bags. A: before DEC treatment, B:15 minutes after exposure to 10µM exposure to DEC . C: after 60 minutes washing APF Ringer at the end of the recordings. Major structures are highlighted.
