# Supplementary material for: Diethylcarbamazine, TRP channels and Ca2+ signaling in cells of the Ascaris intestine
Source: Sci Rep. 2022 Dec 9;12:21317. doi: 10.1038/s41598-022-25648-7 (PMC9734116; doi:10.1038/s41598-022-25648-7)
Supplement: Supplementary file 5 — Supplementary Information 5. [file 41598_2022_25648_MOESM5_ESM.pptx]

## Slide 1
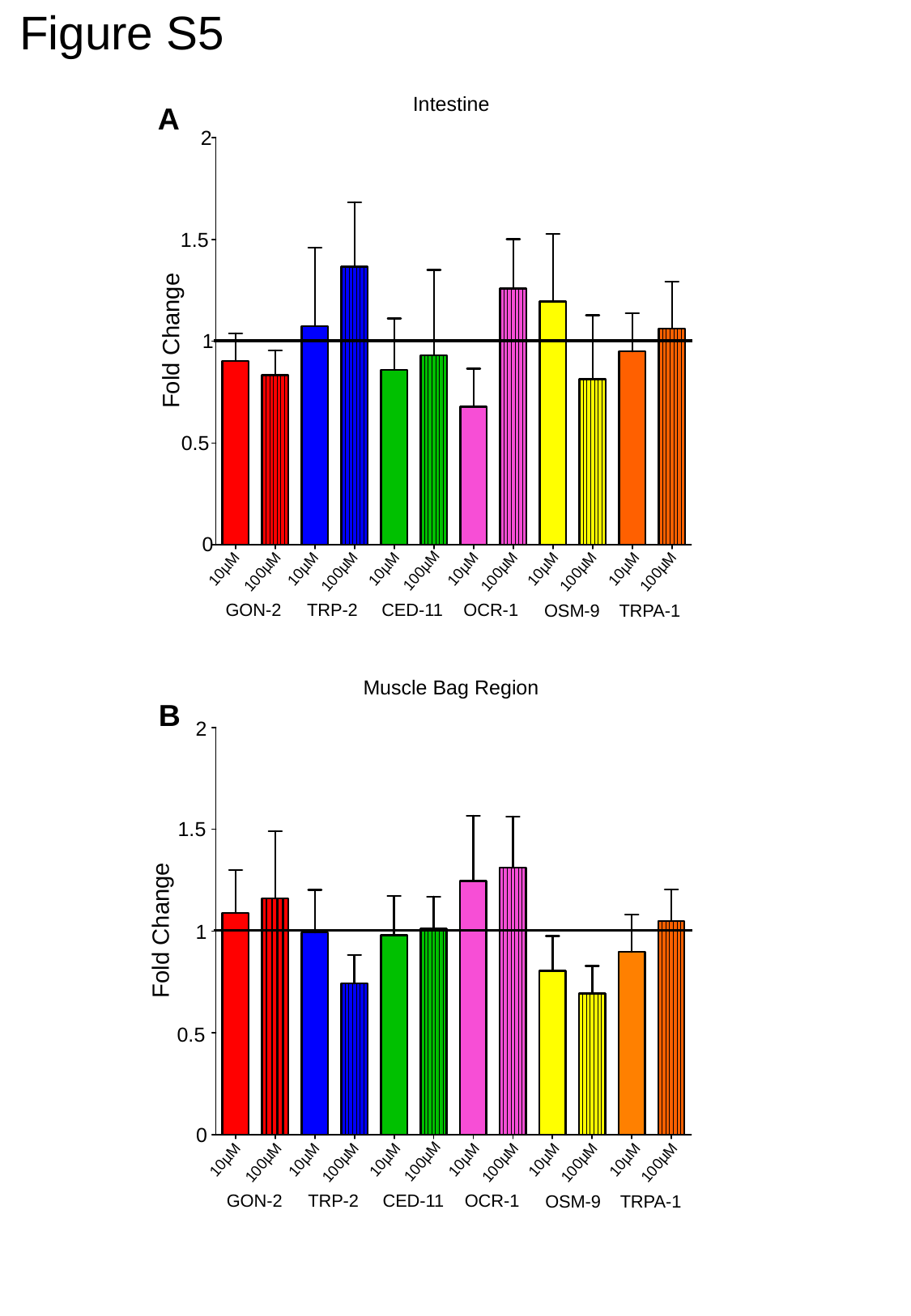

Figure S5
Intestine
2
1.5
1
0.5
0
Fold Change
10µM
10µM
10µM
10µM
10µM
10µM
100µM
100µM
100µM
100µM
100µM
100µM
GON-2
TRP-2
CED-11
OCR-1
TRPA-1
OSM-9
A
Muscle Bag Region
B
2
1.5
1
0.5
0
Fold Change
10µM
10µM
10µM
10µM
10µM
10µM
100µM
100µM
100µM
100µM
100µM
100µM
GON-2
TRP-2
CED-11
OCR-1
TRPA-1
OSM-9
