# Supplementary material for: Diethylcarbamazine, TRP channels and Ca2+ signaling in cells of the Ascaris intestine
Source: Sci Rep. 2022 Dec 9;12:21317. doi: 10.1038/s41598-022-25648-7 (PMC9734116; doi:10.1038/s41598-022-25648-7)
Supplement: Supplementary file 6 — Supplementary Information 6. [file 41598_2022_25648_MOESM6_ESM.docx]

**Supplementary Legends**

**Supplementary Figure 1: Localization of TRP channels in the intestine and muscle bag region.** Original uncropped gel pictures from Fig. 2 showing RT-PCR analysis of intestine (1i, 2i, 3i, 4i, 5i) and muscle bag (1b, 2b, 3b, 4b, 5b) of five separate female *A. suum* worms. Each lane represents the intestine or muscle bag of an individual worm. *Asu-gapdh* from the intestine (Ci) or muscle bag (Cb) was used as a positive control. N.C. = negative control, no cDNA template present. M = FastRuler Middle Range DNA Ladder (ThermoFisher Scientific). A) *Asu-gon-2*, B) *Asu-ced-11* C) *Asu-trp-2*, D) *Asu-ocr-1* E) *Asu-osm-9*, F) *Asu-trpa-1*. Images were taken under UV light with an exposure setting of 3 seconds per 1 frame.

**Supplementary Figure 2:** 1 mM CaCl_2_ APF wash does not stimulate detectable Ca^2+^ signals. A) Representative trace showing long term 1 mM CaCl_2_ APF application followed by 10 mM CaCl_2_ application (grey box). B) Maximal amplitudes of 1 mM CaCl_2_ APF signals (black bar) and 10 mM CaCl_2_ (grey bar). *** Significantly different from 1 mM CaCl_2_ APF ( *P* < 0.0001 *t* = 25.26 *df* = 249; paired *t* – test). N = 5 intestines from 5 individual female *Ascaris.* All values are represented as means ± SEM.

**Supplementary Figure 3:** DEC treatment does not affect enterocyte integrity. Histology pictures of *Ascaris* body sections including the intestine and muscle bags before DEC treatment (A), 15 minutes post DEC application (B), the average peak time of DEC signal and 60 minutes (C) end of recordings. Major structures are highlighted. Images were taken under white light with an exposure setting of 1/350 seconds per 1 frame.

**Supplementary Figure 4: Localization of ORAI-1 and STIM-1 in the intestine and muscle bag region.** Original uncropped gel pictures showing RT-PCR analysis of intestine (1i, 2i, 3i, 4i, 5i) and muscle bag (1b, 2b, 3b, 4b, 5b) of five separate female *A. suum* worms. Each lane represents the intestine or muscle bag of an individual worm. *Asu-gapdh* from the intestine (Ci) or muscle bag (Cb) was used as a positive control. N.C. = negative control, no cDNA template present. M = FastRuler Middle Range DNA Ladder (ThermoFisher Scientific). A) *Asu-orai-1*, B) *Asu-stim-1*. Images were taken under UV light with an exposure setting of 3 seconds per 1 frame.

**Supplementary Figure 5: Long-term DEC exposure does not change TRP channel expression:** A) Transcript level analysis of TRP channels in intestines for GON-2, TRP-2, CED-11, OCR-1, OSM-9, and TRPA-1 after 4-hour treatment with 10 µM DEC (Solid) or 100 µM (lined) when compared to paired untreated intestinal samples. B) Transcript level analysis of TRP channels in muscles bags for GON-2, TRP-2, CED-11, OCR-1, OSM-9, and TRPA-1 after 4-hour treatment with 10 µM DEC (Solid) or 100 µM (lined) when compared to paired untreated muscle samples. One-way analysis of variance (ANOVA) with post-hoc Tukey test, showed no significant difference in transcript levels between 10 µM and 100 µM DEC treated samples in the intestine or muscle bag region. All values represented as means ± SEM.

**Supplementary Table 1.** Primer sequences used for RT-PCR. Forward and reverse primer sequences for the TRP channel genes *gon-2, trp-2, ced-11, ocr-1, osm-9* and *trpa-1*, the calcium channel regulator gene *stim-1*, the store operated calcium channel regulator gene *orai-1* and the reference gene *gapdh* in *Ascaris suum*.

**Supplementary Table 2.** Primer sequences targeting middle of each gene for quantitative PCR. Forward and reverse primers used for qPCR experiments for the TRP channel genes *gon-2, trp-2, ced-11, ocr-1, osm-9* and *trpa-1* and the reference gene *gapdh* in *Ascaris suum*

**Supplementary Table 3:** Accession numbers for genes analyzed for all species used for the generation of the dendrogram Fig.1. N/A = Not Applicable as a suitable gene could not be found in the organisms’ genome or was not compared against other species. Note the two genes listed for *A. suum* OCR-1. Combination of the two genes led to better fit for the tree suggesting that the two genes may be one single gene.
