# Supplementary material for: Diethylcarbamazine, TRP channels and Ca2+ signaling in cells of the Ascaris intestine
Source: Sci Rep. 2022 Dec 9;12:21317. doi: 10.1038/s41598-022-25648-7 (PMC9734116; doi:10.1038/s41598-022-25648-7)
Supplement: Supplementary file 7 — Supplementary Information 7. [file 41598_2022_25648_MOESM7_ESM.docx]

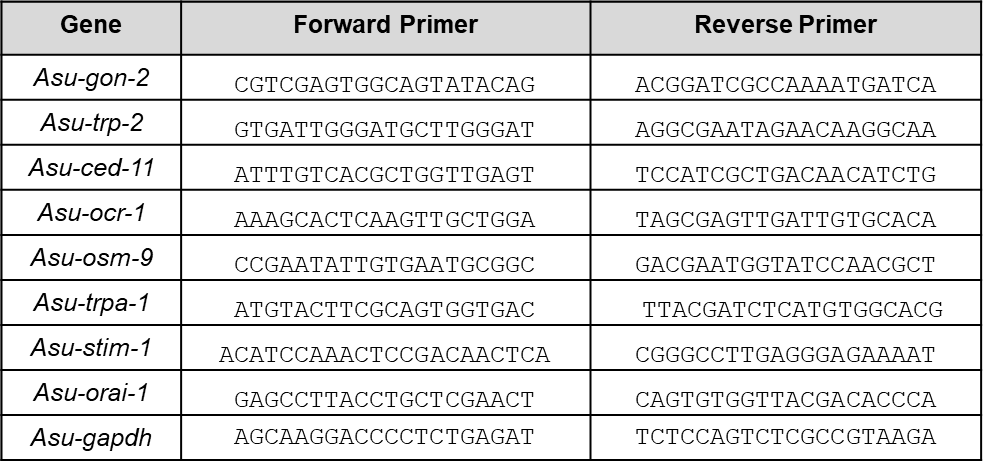
**Supplementary Table S1**

**Supplementary Table S2**


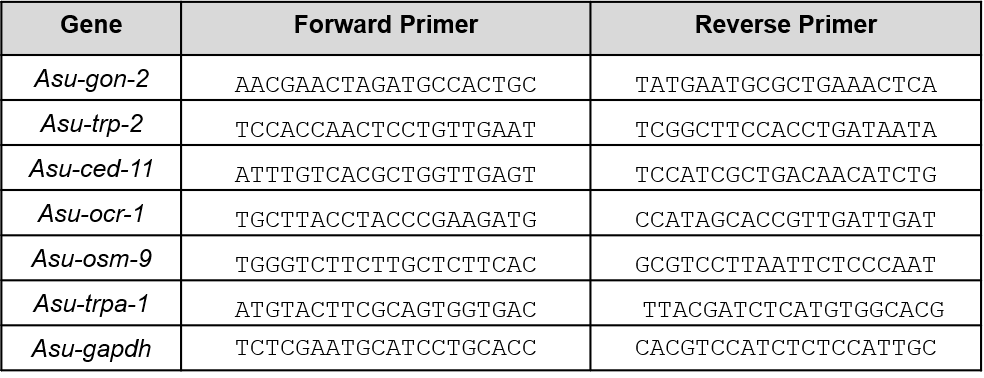


.

| Species | GON-2 | CED-11 | TRP-2 | OCR-1 | OSM-9 | TRPA-1 | STIM-1 | ORAI-1 |
| --- | --- | --- | --- | --- | --- | --- | --- | --- |
| *Ascaris suum* | [**AgR007_g147**](https://parasite.wormbase.org/Ascaris_suum_prjna62057/Gene/Summary?g=AgR007_g147;r=AgR007:2808797-2877783)  [AEUI03000013.1](http://www.ebi.ac.uk/ena/data/view/AEUI03000013.1) | [**AgB05_g111**](https://parasite.wormbase.org/Ascaris_suum_prjna62057/Gene/Summary?g=AgB05_g111;r=AgB05:1973295-1992527)  [AEUI03000007.1](http://www.ebi.ac.uk/ena/data/view/AEUI03000007.1) | [AgR004_g234](https://parasite.wormbase.org/Ascaris_suum_prjna62057/Gene/Summary?g=AgR004_g234;r=AgR004:4316766-4347888)  [AEUI03000009.1](http://www.ebi.ac.uk/ena/data/view/AEUI03000009.1) | [AgR018X_g126](https://parasite.wormbase.org/Ascaris_suum_prjna62057/Gene/Summary?g=AgR018X_g126;r=AgR018X:1825634-1831347) [AgR018X_g125](https://parasite.wormbase.org/Ascaris_suum_prjna62057/Gene/Summary?g=AgR018X_g125;r=AgR018X:1813927-1822884) [AEUI03000026.1](http://www.ebi.ac.uk/ena/data/view/AEUI03000026.1) [AEUI03000026.1](http://www.ebi.ac.uk/ena/data/view/AEUI03000026.1) | [**AgR022_g181**](https://parasite.wormbase.org/Ascaris_suum_prjna62057/Gene/Summary?g=AgR022_g181;r=AgR022:2383235-2399839) [AEUI03000031.1](http://www.ebi.ac.uk/ena/data/view/AEUI03000031.1) | [**AgR022_g092**](https://parasite.wormbase.org/Ascaris_suum_prjna62057/Gene/Summary?g=AgR022_g092;r=AgR022:1069839-1150064)  [AEUI03000031.1](http://www.ebi.ac.uk/ena/data/view/AEUI03000031.1) | [AgR005_g282](https://parasite.wormbase.org/Ascaris_suum_prjna62057/Gene/Summary?db=core;g=AgR005_g282;r=AgR005:4411589-4451489;t=AgR005_g282_t01;tl=MUIxQsfA5nYKIBHf-6186464-155702363)­­  [AEUI03000010.1](http://www.ebi.ac.uk/ena/data/view/AEUI03000010.1) | [AgR021X_g048](https://parasite.wormbase.org/Ascaris_suum_prjna62057/Gene/Summary?db=core;g=AgR021X_g048;r=AgR021X:590583-610345;t=AgR021X_g048_t06;tl=iJfBfgtxhMWHHIpv-6195953-157452972) [AEUI03000029.1](http://www.ebi.ac.uk/ena/data/view/AEUI03000029.1) |
| *Parascaris equorum* | [PEQ_0000273201](https://parasite.wormbase.org/Parascaris_equorum_prjeb514/Gene/Summary?g=PEQ_0000273201;r=PEQ_scaffold0007546:99-5993) [LM470319.1](http://www.ebi.ac.uk/ena/data/view/LM470319.1) | [**PEQ_0001179501**](https://parasite.wormbase.org/Parascaris_equorum_prjeb514/Gene/Summary?g=PEQ_0001179501;r=PEQ_scaffold0000800:2-17333)  [LM463558.1](http://www.ebi.ac.uk/ena/data/view/LM463558.1) | [PEQ_0001228701](https://parasite.wormbase.org/Parascaris_equorum_prjeb514/Gene/Summary?g=PEQ_0001228701;r=PEQ_scaffold0018616:330-2170)  [LM481478.1](http://www.ebi.ac.uk/ena/data/view/LM481478.1) | [PEQ_0001206301](https://parasite.wormbase.org/Parascaris_equorum_prjeb514/Gene/Summary?g=PEQ_0001206301;r=PEQ_scaffold0002058:1274-4079) [LM464818.1](http://www.ebi.ac.uk/ena/data/view/LM464818.1) | [**PEQ_0001023201**](https://parasite.wormbase.org/Parascaris_equorum_prjeb514/Gene/Summary?g=PEQ_0001023201;r=PEQ_scaffold0000479:2187-17945)  [LM463237.1](http://www.ebi.ac.uk/ena/data/view/LM463237.1) | [**PEQ_0001267101**](https://parasite.wormbase.org/Parascaris_equorum_prjeb514/Gene/Summary?g=PEQ_0001267101;r=PEQ_scaffold0001437:1885-13325)  [LM464196.1](http://www.ebi.ac.uk/ena/data/view/LM464196.1) | N/A | N/A |
| *Caenorhabditis elegans* | [WBGene00001651](https://parasite.wormbase.org/Caenorhabditis_elegans_prjna13758/Gene/Summary?g=WBGene00001651;r=I:8549513-8570998)  [BX284601.5](http://www.ebi.ac.uk/ena/data/view/BX284601.5) | [WBGene00006615](https://parasite.wormbase.org/Caenorhabditis_elegans_prjna13758/Gene/Summary?g=WBGene00006615;r=III:991881-1001931)  [BX284603.4](http://www.ebi.ac.uk/ena/data/view/BX284603.4) | [WBGene00006615](https://parasite.wormbase.org/Caenorhabditis_elegans_prjna13758/Gene/Summary?g=WBGene00006615;r=III:991881-1001931) [BX284603.4](http://www.ebi.ac.uk/ena/data/view/BX284603.4) | [WBGene00003838](https://parasite.wormbase.org/Caenorhabditis_elegans_prjna13758/Gene/Summary?g=WBGene00003838;r=V:10764366-10768590)  [BX284605.5](http://www.ebi.ac.uk/ena/data/view/BX284605.5) | [WBGene00003889](https://parasite.wormbase.org/Caenorhabditis_elegans_prjna13758/Gene/Summary?g=WBGene00003889;r=IV:3551090-3557431)  [BX284604.4](http://www.ebi.ac.uk/ena/data/view/BX284604.4) | [WBGene00007801](https://parasite.wormbase.org/Caenorhabditis_elegans_prjna13758/Gene/Summary?g=WBGene00007801;r=IV:11864526-11875733)  [BX284604.4](http://www.ebi.ac.uk/ena/data/view/BX284604.4) | [WBGene00021910](https://parasite.wormbase.org/Caenorhabditis_elegans_prjna13758/Gene/Summary?db=core;g=WBGene00021910;r=III:408127-418738;t=Y55B1BM.1a.1;tl=cpn084oNcZDYd36Z-6195939-157452861) [CCD73858](https://www.ebi.ac.uk/ena/browser/view/CCD73858) | [WBGene00015648](https://parasite.wormbase.org/Caenorhabditis_elegans_prjna13758/Gene/Summary?db=core;g=WBGene00015648;r=III:646754-654402;t=C09F5.2a.1;tl=TrRub4Psqzd3QWDb-6195950-157452910)  [BX284603.4](http://www.ebi.ac.uk/ena/data/view/BX284603.4) |
| *Brugia malayi* | [WBGene00228694](https://parasite.wormbase.org/Brugia_malayi_prjna10729/Gene/Summary?g=WBGene00228694;r=Bm_v4_Chr3_scaffold_001:10221827-10245626)  [CAAKNF010000194.1](http://www.ebi.ac.uk/ena/data/view/CAAKNF010000194.1) | [WBGene00227742](https://parasite.wormbase.org/Brugia_malayi_prjna10729/Gene/Summary?g=WBGene00227742;r=Bm_v4_Chr1_scaffold_001:268425-281730)  [CAAKNF010000192.1](http://www.ebi.ac.uk/ena/data/view/CAAKNF010000192.1) | [WBGene00225507](https://parasite.wormbase.org/Brugia_malayi_prjna10729/Gene/Summary?g=WBGene00225507;r=Bm_v4_Chr1_scaffold_001:14180474-14191510)  [CAAKNF010000192.1](http://www.ebi.ac.uk/ena/data/view/CAAKNF010000192.1) | [WBGene00225952](https://parasite.wormbase.org/Brugia_malayi_prjna10729/Gene/Summary?g=WBGene00225952;r=Bm_v4_Chr4_scaffold_001:6051367-6058391)  [CAAKNF010000195.1](http://www.ebi.ac.uk/ena/data/view/CAAKNF010000195.1) | [WBGene00287459](https://parasite.wormbase.org/Trichuris_muris_prjeb126/Gene/Summary?g=WBGene00287459;r=TMUE_LG2:27435393-27440821)  [CAAKNF010000196.1](http://www.ebi.ac.uk/ena/data/view/CAAKNF010000196.1) | [WBGene00222031](https://parasite.wormbase.org/Brugia_malayi_prjna10729/Gene/Summary?g=WBGene00222031;r=Bm_v4_ChrX_scaffold_001:8218893-8237954)  [CAAKNF010000196.1](http://www.ebi.ac.uk/ena/data/view/CAAKNF010000196.1) | [WBGene00227102](https://parasite.wormbase.org/Brugia_malayi_prjna10729/Gene/Summary?db=core;g=WBGene00227102;r=Bm_v4_Chr1_scaffold_001:8207277-8211720;t=Bm6841a.1;tl=FWcFAJddescixdjr-6195948-157452898)  [CAAKNF010000192.1](http://www.ebi.ac.uk/ena/data/view/CAAKNF010000192.1) | [WBGene00222363](https://parasite.wormbase.org/Brugia_malayi_prjna10729/Gene/Summary?db=core;g=WBGene00222363;r=Bm_v4_Chr1_scaffold_001:2744651-2761972;t=Bm2102a.1;tl=KeO931wTBwxHZDXY-6195952-157452916)  [CAAKNF010000192.1](http://www.ebi.ac.uk/ena/data/view/CAAKNF010000192.1) |
| *Trichuris muris* | [WBGene00291806](https://parasite.wormbase.org/Trichuris_muris_prjeb126/Gene/Summary?g=WBGene00291806;r=TMUE_LG3:8859046-8869258) | [WBGene00291719](https://parasite.wormbase.org/Trichuris_muris_prjeb126/Gene/Summary?g=WBGene00291719;r=TMUE_LG3:11233278-11242054) | [WBGene00291415](https://parasite.wormbase.org/Trichuris_muris_prjeb126/Gene/Summary?g=WBGene00291415;r=TMUE_LG2:27137550-27143967) | [WBGene00287459](https://parasite.wormbase.org/Trichuris_muris_prjeb126/Gene/Summary?g=WBGene00287459;r=TMUE_LG2:27435393-27440821) | [WBGene00290215](https://parasite.wormbase.org/Trichuris_muris_prjeb126/Gene/Summary?g=WBGene00290215;r=TMUE_LG3:11667945-11673881) | [WBGene00289171](https://parasite.wormbase.org/Trichuris_muris_prjeb126/Gene/Summary?g=WBGene00289171;r=TMUE_LG3:16598305-16636296) | N/A | N/A |
| *Schistosoma mansoni* | [Smp_147140](https://parasite.wormbase.org/Schistosoma_mansoni_prjea36577/Gene/Summary?g=Smp_147140;r=SM_V7_6:8957170-9079252)  [LR214935.1](http://www.ebi.ac.uk/ena/data/view/LR214935.1) | [Smp_246790](https://parasite.wormbase.org/Schistosoma_mansoni_prjea36577/Gene/Summary?g=Smp_246790;r=SM_V7_3:646153-793607)  [LR214931.1](http://www.ebi.ac.uk/ena/data/view/LR214931.1) | [Smp_336170](https://parasite.wormbase.org/Schistosoma_mansoni_prjea36577/Gene/Summary?g=Smp_336170;r=SM_V7_4:43281615-43320921)  [LR214933.1](http://www.ebi.ac.uk/ena/data/view/LR214933.1) | N/A | N/A | [Smp_145700](https://parasite.wormbase.org/Schistosoma_mansoni_prjea36577/Gene/Summary?g=Smp_145700;r=SM_V7_5:13192902-13282030)  [LR214934.1](http://www.ebi.ac.uk/ena/data/view/LR214934.1) | N/A | N/A |
| *Drosophila melanogaster* | [A8DYE2](https://www.uniprot.org/uniprot/A8DYE2) | [Q9VJJ7](https://www.uniprot.org/uniprot/Q9VJJ7) | [A0A0B4LGJ2](https://www.uniprot.org/uniprot/A0A0B4LGJ2) | [M9PI57](https://www.uniprot.org/uniprot/M9PI57) | [Q9W3W0](https://www.uniprot.org/uniprot/Q9W3W0) | [Q0KIE7](https://www.uniprot.org/uniprot/Q0KIE7) | N/A | N/A |

**Supplementary Table S3**
